# Increased risk of metabolic disorders in healthy young adults with family history of diabetes: from the Korea National Health and Nutrition Survey

**DOI:** 10.1186/s13098-017-0210-8

**Published:** 2017-03-01

**Authors:** Joon Ho Moon, Eun Roh, Tae Jung Oh, Kyoung Min Kim, Jae Hoon Moon, Soo Lim, Hak Chul Jang, Sung Hee Choi

**Affiliations:** 1 Department of Internal Medicine, Seoul National University College of Medicine, Seoul, South Korea; 2 Department of Internal Medicine, Seoul National University Hospital, Seoul, South Korea; 3 Division of Endocrinology and Metabolism, Asan Medical Center, University of Ulsan College of Medicine, Seoul, South Korea; 4 Department of Internal Medicine, Seoul National University Bundang Hospital, 82, Gumi-ro, 173 Beon-gil, Bundang-gu, Seongnam, Gyeonggi-do 463-707 South Korea; 5 Present Address: Graduate School of Medical Science and Engineering, Korea Advanced Institute of Science and Technology, Daejeon, South Korea

**Keywords:** Family history, Diabetes mellitus, type 2, Metabolic syndrome

## Abstract

**Background:**

We assessed the impact of a family history of diabetes on type 2 diabetes, metabolic syndrome, and behavioral traits in young Korean adults.

**Methods:**

Subjects aged 25–44 years were included, and the presence of a family history of diabetes was obtained by a self-reported questionnaire (the Korea National Health and Nutrition Survey 2010). We compared the prevalence of type 2 diabetes and metabolic syndrome, and other metabolic parameters, including blood pressure and lipid profile.

**Results:**

Of 2059 participants, those with a family history of diabetes involving first-degree relatives (n = 489, 23.7%) had a significantly higher prevalence of impaired fasting glucose (14.3 vs. 11.7%) and type 2 diabetes (6.7 vs. 1.8%), compared to those without a family history (*P* < 0.001). The prevalence of metabolic syndrome (21.3 vs. 12.1%, *P* < 0.001) and its components (except for high-density lipoprotein cholesterol) were greater in subjects with a family history of diabetes. Among subjects exhibiting normal glucose tolerance (n = 1704), those with a family history of diabetes had higher fasting glucose (89.0 vs. 87.8 mg/dL, *P* < 0.001) and triglyceride (100.5 vs. 89.0 mg/dL, *P* < 0.001), and lower beta cell function by the homeostasis model assessment (HOMA-β; 134.2 vs. 137.5, *P* = 0.020). The obesity indices (body mass index, waist circumference, and triglyceride) were significantly correlated with those of both parents (*P* < 0.01 for all variables). Risk-reducing behavior, including regular exercise (18.2 vs. 19.7%, *P* = 0.469) and calorie intake (2174.8 vs. 2149.1 kcal/day, *P* = 0.636), did not markedly differ according to a family history of diabetes.

**Conclusions:**

Young adults with a family history of diabetes had an increased risk of type 2 diabetes and metabolic syndrome, even though they currently exhibited a normal glycemic profile. Proactive lifestyle consultation is requested especially among healthy young population with a family history of diabetes.

**Electronic supplementary material:**

The online version of this article (doi:10.1186/s13098-017-0210-8) contains supplementary material, which is available to authorized users.

## Background

The prevalence of diabetes has increased worldwide during recent decades [1, 2]. The global estimate of people with diabetes is expected to increase to 592 million by 2035 [2]; however, a high proportion of individuals with diabetes (up to 174.8 million) remain undiagnosed [3]. It is clinically important to identify individuals at risk of diabetes and metabolic disorders to prevent long-term complications and to reduce the socioeconomic burden of diabetes.

Young adults with metabolic risk factors such as obesity and family history of diabetes have concerns regarding the potential for future development of chronic metabolic diseases. The early detection of such young adults with a future risk of metabolic disorders is vital, as the progression to diabetes become irreversible after a certain stage [4]. The clinical practice guidelines of the American Diabetes Association recommend that diabetes screening should be initiated at 45 years of age, particularly among individuals who are overweight [5]; hence, young adults (those aged <45 years) were disregarded from these screening programs. Nevertheless, we believe that young adults should be stratified according to their risk of future diabetes, and the high-risk population should undergo regular screening.

The development of diabetes mellitus is based on multiple factors. In particular, the assessment of the family history is an inexpensive and useful tool that can reflect both the genetic and environmental factors shared by families [6]. In fact, a family history of diabetes is found to be associated with both cases of insulin secretory defect [7] and insulin resistance [8]. According to previous studies conducted among Caucasians, the incidence of type 2 diabetes was increased by 1.4- to 6.1-fold among individuals with a family history of diabetes [6, 9–11]. Although the increased metabolic risk of having a family history of diabetes is expected to be similar among Asians, its attributable risk might vary due to different genetic and environmental background [12]. In a cross-sectional study of 46,239 middle-aged Chinese men and women (mean age, 44.9 years), the familial risk of diabetes exhibited a graded association with the prevalence of diabetes (6.16-fold higher prevalence if two generations of first-degree relatives have diabetes, and 2.86-fold if one generation of first-degree relatives have diabetes) [13]. Middle-aged Japanese men and women (mean age 46.2 years) had 1.8-fold increase in incident diabetes among whom had a family history of diabetes in a 7-year follow-up study [14]. In Korea, some studies have described the odds ratio (ORs) for type 2 diabetes in middle-aged individuals with a family history of diabetes (i.e. OR = 2.59 in a study by Kim et al. [15] and OR = 1.86 by Lee et al. [16]); however, family history was assessed only as a component of multiple risk factors. To conclude, studies conducted among Asian population revealed similarly increased risk of diabetes than non-Asians among subjects with a family history of diabetes, but none of these studies particularly focused on young adults who should be meticulously evaluated for future risk of metabolic disease.

In the present study, we aimed to (1) evaluate the family history of diabetes as an independent risk factor for type 2 diabetes and metabolic syndrome; (2) compare the metabolic parameters according to family history in young adults with currently normal glucose levels; (3) identify the metabolic parameters that are most likely to be inherited to offspring; and (4) assess the metabolic risk-reducing behaviors by presence of a family history of diabetes, among young Korean adults by using nationwide representative survey data.

## Methods

### Data source and study population

The data analyzed in the present study were obtained from the Korea National Health and Nutrition Survey (KNHANES) V (2010). The KNHANES is a nationwide community-based cross-sectional survey that examined the general health and nutritional status of non-institutionalized civilians in Korea. The KNHANES comprised 3 distinct surveys: health interview survey, health examination survey, and nutrition survey. Participants were selected from sampling units based on the geographical area, sex, and age groups. The KNHANES was conducted according to the guidelines specified in the Declaration of Helsinki. The Institutional Review Board of the Korean Centers for Disease Control and Prevention approved the study protocol (IRB number: 2010-02CON-21-C).

Subjects who were aged 25–44 years and completed the health interview and examination survey were eligible for the current analysis (n = 2218). We limited our study population to age <45 to evaluate the metabolic risk of a family history of diabetes among young adults who are usually not indicated for diabetes screening [12]. To minimize the number of subjects with potential type 1 diabetes in the study population, we set a lower age cutoff of 25 years, after which the incidence of type 1 diabetes is shown to clearly decrease [17]. Subjects who were diagnosed with diabetes prior to 25 years of age or those who had started insulin treatment within 1 year of diabetes diagnosis were also excluded (n = 2). Moreover, subjects with pregnancy, cancer (except thyroid cancer), and steroid medication were excluded for the analysis (n = 159). A subgroup of the subjects’ parents participated in the KNHANES (n = 578, 28.1%); these individuals were administered the same survey.

### Definition of family history

A family history of diabetes involving first-degree relatives (parents and siblings) was recorded via the self-reported questionnaire in the health interview survey. By utilizing parents’ self-report as the standard, the estimated sensitivity, specificity, and accuracy of proband-reported family history in this study was 84.3, 97.0, and 95.2%. The validity of family history was generally in accordance with previous studies [18–21].

### Definition of type 2 diabetes and metabolic syndrome

The primary outcome of the study was the prevalence of type 2 diabetes and metabolic syndrome by a family history of diabetes. Diagnosis of diabetes, impaired fasting glucose (IFG), and normal glucose tolerance (NGT) were based on the American Diabetes Association guidelines [5]. Abnormal glucose tolerance (AGT) was considered in cases with either IFG or diabetes. Metabolic syndrome was diagnosed if ≥3 of the criteria were met, according to the National Cholesterol Education Program (NCEP) Adult Treatment panel (ATP) III revised criteria [22]. Central obesity was defined as waist circumference (WC) >90 cm in men and >80 cm in women, based on the International Obesity Task Force criteria for the Asian–Pacific population [23]. Metabolically healthy status was either defined by (1) the absence of metabolic syndrome (<3 components of metabolic syndrome) [24], or (2) high insulin sensitivity [defined by homeostasis model assessment of insulin resistance (HOMA-IR) <2.5] [25].

### Anthropometric and biochemical assessments

Height and WC was measured to the nearest 0.1 cm and weight was measured to the nearest 0.1 kg. Blood pressure was measured twice with a mercury sphygmomanometer (Baumanometer, Baum, Copiague, NY, U.S.A.), with the patient in the sitting position after a 10-min rest. An averaged value of these measurements was used for the analysis.

Blood samples were drawn from the antecubital vein in the morning after fasting for at least 8 h. The fasting plasma concentrations of glucose, total cholesterol, triglyceride, high-density lipoprotein (HDL) cholesterol, aspartate transaminase, alanine transaminase, and gamma-glutamyl transpeptidase were measured enzymatically using an autoanalyzer (Hitachi 7600, Hitachi, Tokyo, Japan). Insulin was measured by immunoradiometric assay (1470 WIZARD gamma-Counter, PerkinElmer, Turku, Finland). Glycated hemoglobin was measured by high-performance liquid chromatography (HLC-723G7, Tosoh, Tokyo, Japan). The HOMA value of insulin resistance (HOMA-IR) and β-cell function (HOMA-β) were also calculated [26]. The level of low-density lipoprotein (LDL) cholesterol was calculated using Friedewald’s formula [27].

### Exercise and diet

Physical activity data were collected from the self-administered questionnaire; physical activity was classified as mild, moderate, or vigorous [28]. Regular exercise was defined as moderate intensity activity for >30 min, for 5 times a week, or vigorous intensity activity for >20 min, for 3 times a week. Dietary data were collected using the food intake questionnaire, which is an open-ended survey for reporting food consumption via the 24-h recall method [29].

### Statistical analysis

Data are presented as number (%), mean ± standard deviation for the variables in a Gaussian distribution, or as median (interquartile range) for the other variables. The χ^2^ test was used for comparing categorical variables. ORs were estimated with their corresponding 95% confidence intervals (CIs), as well as *P* values, to evaluate the risk of type 2 diabetes and metabolic syndrome according to the family history of diabetes. Sensitivity analysis according to body mass index (BMI) (normal weight: BMI < 23 kg/m^2^, overweight: 23 ≤ BMI < 25 kg/m^2^, obese: BMI ≥ 25 kg/m^2^) was performed. Statistical differences between the groups were examined using Student’s *t* test or analysis of variance (ANOVA) for continuous variables. Variables with a skewed distribution were log-transformed for analyses. Variables that showed significant association with a family history of diabetes were further adjusted for age and BMI. Correlation analysis was conducted for BMI, WC, fasting plasma glucose (FPG), triglyceride, HOMA-IR, and HOMA-β between the parents and offspring. Statistical significance was defined as a *P* value of <0.05. All statistical analyses were performed using SPSS version 20.0 (IBM Co., Armonk, NY, U.S.A.).

## Results

### Baseline characteristics

A total of 2059 subjects (876 men and 1183 women) aged 25–44 years were included in the analysis. The baseline characteristics of all study participants according to the glycemic status are described in Additional file 1: Table S1. The mean age was 35.5 ± 5.4 years and mean BMI was 23.4 ± 3.6 kg/m^2^. Of these, 1744 (84.7%) exhibited NGT, 254 exhibited IFG (12.3%), and 61 (3.0%) exhibited type 2 diabetes. The men had a higher prevalence of AGT (21.2%, 185 subjects) than the women (11.0%, 130 subjects) (*P* < 0.001). Subjects with IFG or type 2 diabetes exhibited adverse metabolic parameters, including BMI, blood pressure, FPG concentration, lipid profile, HOMA-IR, and HOMA-β in both genders, compared to those with NGT.

Of all the subjects, 489 (23.7%) had at least 1 first-degree relative with diabetes (men, 221 [25.2%]; women, 268 [22.7%]). Among these subjects, 476 (23.1%) had at least one parent with diabetes, including 255 (12.4%) who had a father with diabetes, 173 (8.4%) who had a mother with diabetes, and 48 (2.3%) who had both parents with diabetes. Forty subjects (1.9%) had a sibling with diabetes. A total of 421 (20.4%) had 1 family member with diabetes, 61 (3.0%) had 2 family members with diabetes, and 7 (0.3%) had 3 family members with diabetes.

### Glycemic status and prevalence of metabolic syndrome according to the family history of diabetes

**Fig. 1:**
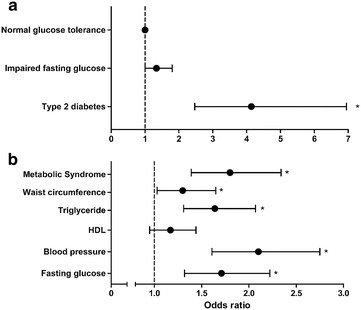
Odds ratios for type 2 diabetes and metabolic syndrome in subjects with family history of diabetes. Risks for impaired fasting glucose, type 2 diabetes (**a**), metabolic syndrome and each component (**b**) in subjects with family history of diabetes in first-degree relatives are presented by odds ratio. Criteria for metabolic syndrome followed NCEP-ATP III revised criteria. *P < 0.05

We then evaluated the glycemic status and prevalence of metabolic syndrome according to the family history of diabetes involving first-degree relatives. Among 489 subjects with a family history of diabetes, 386 (78.9%) exhibited NGT, 70 (14.3%) exhibited IFG, and 33 (6.7%) exhibited type 2 diabetes, with increasing trend compared to those without a family history of diabetes (*P*-for-trend <0.001) (Table 1). The OR for IFG among subjects with a family history of diabetes was 1.34 (95% CI 1.00–1.80), whereas that for type 2 diabetes among subjects with a family history of diabetes was 4.14 (95% CI 2.47–6.95) (Fig. 1a).

**Table 1:** Glycemic status and prevalence of metabolic syndrome according to family history of diabetes in first-degree relatives

	Total (n = 2059)			Men (n = 876)			Women (n = 1183)		
	FH−	FH+	*P*	FH−	FH+	*P*	FH−	FH+	*P*
	(n = 1570)	(n = 489)		(n = 655)	(n = 221)		(n = 915)	(n = 268)	
*Glycemic status*									
NGT	1358 (86.5%)	386 (78.9%)	<*0.001*	533 (81.4%)	158 (71.5%)	<*0.001*	825 (90.2%)	228 (85.1%)	*0.019*
IFG	184 (11.7%)	70 (14.3%)		111 (16.9%)	42 (19.0%)		73 (8.0%)	28 (10.4%)	
Diabetes	28 (1.8%)	33 (6.7%)		11 (1.7%)	21 (9.5%)		17 (1.9%)	12 (4.5%)	
*Prevalence of metabolic syndrome*	190 (12.1%)	104 (21.3%)	<*0.001*	123 (18.8%)	74 (33.5%)	<*0.001*	67 (7.3%)	30 (11.2%)	*0.042*
Waist circumference (>90 cm in men and >80 cm in women)	344 (22.1%)	131 (26.9%)	*0.029*	144 (22.1%)	58 (26.5%)	0.186	200 (22.1%)	73 (27.2%)	0.082
Triglycerides (≥150 mg/dL or medication use)	323 (20.6%)	146 (29.9%)	<*0.001*	236 (36.1%)	105 (47.5%)	*0.003*	87 (9.5%)	41 (15.3%)	*0.010*
HDL cholesterol (<40 mg/dL in men and <50 mg/dL in women or medication use)	583 (37.2%)	200 (40.9%)	0.142	205 (31.3%)	83 (37.6%)	0.089	378 (41.4%)	117 (43.7%)	0.511
Blood pressure (≥130/85 mmHg or antihypertensive medication use)	222 (14.1%)	100 (20.4%)	*0.001*	162 (24.7%)	83 (37.6%)	<*0.001*	60 (6.6%)	17 (6.3%)	0.901
Fasting glucose (≥100 mg/dL or medication use)	212 (13.5%)	103 (21.1%)	<*0.001*	122 (18.6%)	63 (28.5%)	*0.002*	90 (9.8%)	40 (14.9%)	*0.019*

Statistical significance was defined as a *P* value of <0.05 (Italic values)

*FH* family history of diabetes, *IFG* impaired fasting glucose, *NGT* normal glucose tolerance

*P* values are from χ^2^-test

We also assessed the association between metabolic syndrome and the family history of diabetes. The prevalence of metabolic syndrome was 21.3% (n = 104 of 489) among subjects with a family history of diabetes, which was significantly higher than 12.1% (n = 190 of 1570) among subjects without a family history of diabetes (*P* < 0.001). Each component of metabolic syndrome, except for HDL level, showed higher prevalence in subjects with a family history of diabetes (Fig. 1b). We further analyzed metabolically healthy status (<3 components of metabolic syndrome) in combination with obesity [24]. Subjects with a family history of diabetes tend to have lower proportion of metabolically healthy non-obese, but higher proportion of metabolically unhealthy non-obese and metabolically unhealthy obese (Additional file 1: Table S2). The degree of insulin sensitivity is also a frequently used measure to define metabolically healthy status [25]. A total of 40.3% of subjects with a family history of diabetes had insulin resistance (defined by HOMA-IR ≥2.5), whereas 31.0% of subjects without a family history of diabetes had insulin resistance (HOMA-IR; 2.60 ± 1.49 vs. 2.34 ± 1.30, *P* = 0.001) (Additional file 1: Table S2).

We performed stratified analysis according to BMI status, as obesity may contribute to increased risk of metabolic disease in subjects with family history of diabetes. In fact, subjects with a family history had higher BMI subjects without a family history of diabetes (23.7 ± 3.6 vs. 23.1 ± 3.5 kg/m^2^, *P* = 0.001). In each BMI group, subjects with a family history had higher prevalence of AGT (either IFG or diabetes) and metabolic syndrome compared to subjects without family history (Additional file 1: Table S3); only exception in AGT of overweight group did not reach statistical significance (*P* = 0.107). To conclude, an increased risk of metabolic disease in subjects with a family history of diabetes was consistent in different BMI groups.

### Metabolic profiles of young adults with NGT according to the family history of diabetes

We evaluated the metabolic parameters among young adults with currently normal glucose tolerance but had a family history of diabetes (Table 2). We initially adjusted for age, as the subjects with a family history of diabetes were older. Subjects with a family history of diabetes had higher BMI, diastolic blood pressure, total cholesterol, triglyceride, and FPG, and lower HOMA-β. After further adjustment for BMI, triglyceride and FPG remained higher, and HOMA-β remained lower in subjects with a family history of diabetes. A similar trend was observed when IFG subjects were included in the analysis; in fact, the triglyceride and FPG levels were still higher in subjects with a family history of diabetes after these adjustments (Additional file 1: Table S4).

**Table 2:** Clinical characteristics of subjects with NGT according to family history of diabetes in first-degree relatives

	Total (n = 1704)					Men (n = 691)					Women (n = 1053)				
	FH−	FH+	*P*	*P* ^a^	*P* ^b^	FH−	FH+	*P*	*P* ^a^	*P* ^b^	FH−	FH+	*P*	*P* ^a^	*P* ^b^
	(n = 1358)	(n = 346)				(n = 533)	(n = 158)				(n = 825)	(n = 228)			
Age (years)	34.8 ± 5.4	35.7 ± 5.2	*0.004*			35.1 ± 5.5	35.5 ± 5.5	0.439			34.7 ± 5.3	35.9 ± 5.0	*0.002*		
Height (cm)	165.0 ± 8.4	165.5 ± 8.3	0.320	0.200	0.320	172.9 ± 5.7	173.0 ± 5.5	0.887	0.767	0.766	160.0 ± 5.4	160.4 ± 5.5	0.301	0.113	0.107
Weight (cm)	62.2 ± 11.9	63.8 ± 12.2	*0.023*	*0.027*	0.286	71.5 ± 10.4	73.6 ± 10.8	*0.025*	*0.020*	0.845	56.3 ± 8.7	57.0 ± 7.7	0.242	0.832	0.108
BMI (kg/m^2^)	22.7 ± 3.3	23.2 ± 3.3	*0.023*	*0.046*		23.9 ± 3.1	24.6 ± 3.4	*0.018*	*0.012*		22.0 ± 3.2	22.2 ± 2.8	0.443	0.853	
WC (cm)	77.1 ± 9.6	78.1 ± 9.3	0.058	0.108	0.944	82.4 ± 8.6	84.0 ± 8.5	*0.035*	*0.039*	0.806	73.6 ± 8.6	74.0 ± 7.6	0.492	0.774	0.778
SBP (mmHg)	107.9 ± 12.5	109.2 ± 12.4	0.062	0.126	0.331	113.0 ± 12.1	114.8 ± 11.5	0.085	0.096	0.291	104.6 ± 11.7	105.4 ± 11.5	0.380	0.766	0.776
DBP (mmHg)	71.4 ± 10.1	72.8 ± 10.0	*0.019*	*0.045*	0.153	75.8 ± 10.2	78.0 ± 10.0	*0.017*	*0.021*	0.095	68.6 ± 9.0	69.2 ± 8.4	0.359	0.713	0.724
TC (mg/dL)	179.9 ± 33.4	184.9 ± 32.4	*0.009*	*0.024*	0.086	186.4 ± 34.4	195.3 ± 35.9	*0.006*	*0.007*	*0.034*	175.9 ± 32.1	177.5 ± 27.8	0.476	0.641	0.667
TG (mg/dL)^c^	89.0 (62.0–134.0)	100.5 (65.0–162.8)	<*0.001*	<*0.001*	*0.002*	112.0 (77.5–165.0)	137.0 (92.8–203.0)	<*0.001*	<*0.001*	*0.006*	72.0 (53.5–104.0)	78.0 (55.0–109.8)	*0.020*	*0.022*	*0.019*
HDL (mg/dL)^c^	48.7 (41.7–56.5)	46.9 (40.8–56.5)	0.085	0.147	0.367	45.2 (40.0–52.2)	43.4 (38.2–49.8)	0.064	0.070	0.277	52.2 (45.2–60.0)	52.6 (44.3–59.1)	0.524	0.823	0.835
LDL (mg/dL)	108.6 ± 29.1	110.9 ± 26.9	0.164	0.314	0.576	113.3 ± 30.6	117.8 ± 29.0	0.113	0.136	0.313	105.7 ± 27.7	106.3 ± 24.5	0.779	0.887	0.871
AST (IU/L)^c^	18.0 (15.0–21.0)	18.0 (16.0–22.0)	0.467	0.604	0.993	20.0 (18.0–24.5)	22.0 (18.0–26.0)	0.205	0.211	0.556	16.0 (15.0–19.0)	16.0 (15.0–18.0)	0.476	0.276	0.270
ALT (IU/L)^c^	14.0 (11.0–22.0)	15.0 (11.0–23.0)	0.076	0.108	0.338	21.0 (15.0–31.0)	24.0 (17.8–32.3)	*0.037*	*0.037*	0.244	12.0 (10.0–15.0)	12.0 (10.0–15.8)	0.715	0.974	0.991
GGT (IU/L)^c^	17.0 (12.0–27.5)	18.0 (13.0–30.0)	*0.033*	0.062	0.171	29.0 (19.0–48.0)	30.0 (22.0–53.0)	0.107	0.132	0.460	13.0 (11.0–17.0)	14.0 (12.0–18.0)	0.110	0.137	0.138
FPG (mg/dL)	87.8 ± 5.9	89.0 ± 5.5	<*0.001*	*0.003*	*0.006*	89.1 ± 5.8	90.0 ± 5.2	0.094	0.115	0.327	87.0 ± 5.8	88.3 ± 5.4	*0.002*	*0.009*	*0.008*
Insulin (μIU/mL)^c^	9.25 (7.61–11.40)	9.27 (7.69–11.78)	0.830	0.720	0.667	9.32 (7.56–11.45)	9.76 (7.89–12.42)	0.115	0.104	0.602	8.72 (7.58–11.53)	9.16 (7.63–11.36)	0.272	0.335	0.265
HOMA-IR^c^	2.00 (1.63–2.53)	2.05 (1.66–2.64)	0.391	0.367	0.877	2.06 (1.65–2.58)	2.24 (1.77–2.68)	0.074	0.071	0.486	1.92 (1.59–2.48)	1.96 (1.61–2.47)	0.668	0.685	0.606
HOMA-β^c^	137.5 (111.1–174.6)	134.2 (105.0–164.9)	*0.020*	*0.047*	*0.018*	130.8 (105.6–165.4)	135.50 (103.68–170.87)	0.745	0.655	0.787	140. 7 (114.1–179.0)	132.6 (105.2–163.8)	*0.001*	*0.006*	*0.004*

Subjects with normal glucose tolerance are included in this analysis (n = 1704). Data are presented as mean ± standard deviation (for normal distribution) or as median (interquartile range)

Statistical significance was defined as a *P* value of <0.05 (Italic values)

*FH* family history of diabetes, *WC* waist circumference, *SBP* systolic blood pressure, *DBP* diastolic blood pressure, *TC* total cholesterol, *TG* triglycerides, *FPG* fasting plasma glucose, *HOMA*-*IR and HOMA*-*B* homeostatic model assessment of insulin resistance and beta cell

*P* values are calculated by *t* test

^a^ *P* values adjusted for age

^b^ *P* values adjusted for age and BMI

^c^These variables were log transformed for the analysis

### Number of family members with diabetes and adverse metabolic outcome

We then evaluated whether the number of family members with diabetes affected the prevalence of AGT and metabolic syndrome **(**Additional file 1: Table S5**)**. The prevalence of AGT was higher when they had more family members with diabetes. A total of 13.5% of subjects without a family history of diabetes had AGT, whereas 20.4% of subjects with 1 family member with diabetes and 25.0% of subjects with ≥2 family members with diabetes, had AGT. The prevalence of metabolic syndrome also increased as the number of family members with diabetes increased (no family history of diabetes, 12.0%; 1 family member with diabetes, 19.7%; ≥2 family members with diabetes, 27.9%).

### Correlation of metabolic parameters between parents and offspring

To identify the metabolic parameters that were inherited or had a strong correlation with those of their parents, we compared the BMI, WC, FPG, triglyceride levels, HOMA-IR, and HOMA-β between parents and their offspring via a correlation analysis (Table 3). The BMI, WC, and triglyceride concentration of the participants were significantly correlated with those of both parents, whereas the FPG concentration and HOMA-β were only correlated with those of the mother.

**Table 3:** Correlation of metabolic profiles between parents and their offspring

	Pearson’s coefficient	*P*
*Body mass index*		
Father	0.127	*0.029*
Mother	0.214	<*0.001*
*Waist circumference*		
Father	0.152	*0.009*
Mother	0.233	<*0.001*
*Fasting plasma glucose*		
Father	0.051	0.398
Mother	0.122	*0.033*
*Triglyceride*		
Father	0.197	*0.001*
Mother	0.173	*0.002*
*HOMA*-*IR*		
Father	0.083	0.172
Mother	0.041	0.480
*HOMA*-*β*		
Father	0.116	0.055
Mother	0.128	*0.025*

Pearson’s correlation efficient between parents and their offspring are presented (n = 578). HOMA-IR and HOMA-β were log transformed for the analysis

Statistical significance was defined as a *P* value of <0.05 (Italic values)

### Risk-reducing behavior and diabetes status according to family history of diabetes

We analyzed the risk-reducing behavior and diabetes status in subjects with a family history of diabetes (Additional file 1: Table S6). The proportion of subjects who performed regular exercise (with vs. without a family history of diabetes; 18.2% vs. 19.7%) and those who attempted to lose weight (73.0 vs. 74.5%) did not differ according to the family history of diabetes. The total energy intake (2175.8 vs. 2149.1 kcal) and macronutrient consumption were similar between the two groups. Among the subjects with diabetes (n = 61), the diabetes recognition rate (63.6 vs. 57.1%, *P* = 0.605) and treatment rate (42.4 vs. 32.1%, *P* = 0.409) did not significantly differ between the 2 groups. However, the FPG concentration was higher among subjects with a family history of diabetes (170.9 ± 73.9 vs. 140.3 ± 46.3 mg/dL, *P* = 0.050), which suggests the poor diabetes control. The prevalence of diabetic retinopathy was also higher in subjects with a family history of diabetes, although the difference was not significant (18.2 vs. 10.7%, *P* = 0.412).

## Discussion

In the present study, we observed that the prevalence of type 2 diabetes and metabolic syndrome was greater in young Korean adults (aged 25–44 years) with a family history of diabetes, based on a nationwide representative survey. Moreover, young adults with a currently normal glucose tolerance, but has family history of diabetes, had higher FPG and triglyceride levels, which indicates a future risk of progression to type 2 diabetes and metabolic disorders. In addition, the obesity-related parameters, including BMI, WC, and triglyceride concentration, were significantly correlated with those of the parents. However, the risk-reducing behavior, including exercise and calorie intake, did not markedly differ according to the family history of diabetes.

The family history of diabetes appears to be an inexpensive and promising health tool to estimate the public metabolic risk, and is reportedly associated with adverse metabolic outcomes such as type 2 diabetes and atherosclerotic cardiovascular disease [11, 30–32]. The incidence of type 2 diabetes increased by 1.4- to 6.1-fold in the cases with a family history of diabetes; the specific value differed according to study design, definition, and demographic characteristics [6]. Young adults aged <45 years were enrolled in the present study, whereas previous studies primarily included middle-aged adults or those of all ages [9–11, 13–16]. The American Diabetes Association suggested that diabetes screening should begin at the age of 45 years, particularly among obese individuals [5]; however, young adults should also be considered for screening depending on the risk factors. We observed a higher prevalence of type 2 diabetes and metabolic syndrome, along with deteriorated metabolic profiles including FPG levels, triglyceride levels, and HOMA-β, even in young adults with good glucose tolerance but with a family history of diabetes. Notably, family history of diabetes itself was associated with an increased BMI in our analysis. Hence, we explored the strong correlation of BMI, WC, and triglyceride concentration between parents and their offspring. Young adults who are expected to have a higher risk of developing metabolic disorders (*i.e.* those with multiple family members with diabetes and those who are obese) should be considered for regular screening for diabetes even though they may currently have a normal metabolic profile.

Lifestyle modifications and close monitoring for diabetes should be encouraged in subjects at risk of metabolic disorders. In the HealthStyles 2004 survey, the presence of a family history of diabetes was positively associated with risk awareness and risk-reducing behaviors in adults in the United States [10]. In contrast, Korean adults reported a lower perceived risk of developing diabetes as compared to Caucasians [33]. In the present study, no significant difference in the risk-reducing behavior, including exercise and diet, was observed in subjects with a family history of diabetes. Hence, healthcare providers should attempt to educate subjects with a family history of diabetes regarding the need for lifestyle changes and better awareness of the metabolic risk, particularly among ethnicities with a lower perceived risk.

Our study has several distinctive features. First, we included young adults aged <45 years who had a relatively lower risk of metabolic disorders and were neglected for diabetes screening based on the clinical guidelines. To our knowledge, this is one of the first studies to comprehensively assess the risk of metabolic disease and behavioral patterns particularly among young adults. We propose that the risk of type 2 diabetes and metabolic syndrome is greater among young adults (aged <45 years) with a family history of diabetes, and suggest that screening and lifestyle interventions are needed. Second, we included both parents and their progeny as a cluster, which facilitated the correlation analysis of various metabolic parameters, in order to determine the inheritance of obesity.

Our study has several limitations. First, due to the cross-sectional nature of the study, we could not investigate the causal relationship or its underlying mechanism. In addition, several confounding factors might have contributed to our results. For example, BMI was higher among subjects with a family history of diabetes, but these differences did not affect the main purpose of this study, as it suggests that a family history of diabetes itself is associated with an increased risk of obesity and its complications. In addition, we performed additional analyses, by stratifying for age and BMI, to control for these parameters. Second, recall bias might have contributed to the results, as the questionnaires were self-administered. Hence, we validated the family history collected by the questionnaire, and found that the accuracy was as high as 95.2%. Third, subjects with type 1 diabetes might have been included in the study population; nevertheless, we attempted to exclude these subjects by limiting the age range from 25 to 44 years.

## Conclusion

By assessing the nationwide survey data representing the Korean population, we found that a family history of diabetes was associated with an increased risk of metabolic disorders in young adults. Hence, young adults with diabetes risk factors, such as a family history of diabetes, should be considered for screening of diabetes and metabolic disorders. We advocate that family history assessment—an inexpensive but precious measure—should be included as a public health screening tool. Further studies should focus on defining specific criteria for diabetes screening, such as age range, test measure, and the interval to effectively and efficiently detect persons at risk.

## Additional file

**Additional file 1: Table S1.** Baseline characteristics and family history of study subjects by glycemic status. **Table S2.** Metabolic healthy status by family history of diabetes. **Table S3.** Subgroup analysis according to BMI. **Table S4.** Clinical characteristics of non-diabetic subjects according to family history of T2DM in first-degree relatives. **Table S5.** Number of family members with diabetes and the risk of abnormal glucose tolerance and metabolic syndrome. **Table S6.** Exercise and dietary pattern by family history of diabetes.
