# Supplementary material for: Increased risk of metabolic disorders in healthy young adults with family history of diabetes: from the Korea National Health and Nutrition Survey
Source: Diabetol Metab Syndr. 2017 Mar 1;9:16. doi: 10.1186/s13098-017-0210-8 (PMC5333414; doi:10.1186/s13098-017-0210-8)
Supplement: Supplementary file 1 — Additional file 1: Table S1. Baseline characteristics and family history of study subjects by glycemic status. Table S2. Metabolic healthy status by family history of diabetes. Table S3. Subgroup analysis according to BMI. Table S4. Clinical characteristics of non-diabetic subjects according to family history of T2DM in first-degree relatives. Table S5. Number of family members with diabetes and the risk of abnormal glucose tolerance and metabolic syndrome. Table S6. Exercise and dietary pattern by family history of diabetes. [file 13098_2017_210_MOESM1_ESM.docx]

**SUPPLEMENTAL MATERIALS**

**Supplemental Table 1.** Baseline characteristics and family history of study subjects by glycemic status

|  | **Total (n = 2,059)** | | | | | **Men (n = 876)** | | | | **Women (n = 1,183)** | | | |
| --- | --- | --- | --- | --- | --- | --- | --- | --- | --- | --- | --- | --- | --- |
|  | **NGT**  **(n = 1,744)** | **IFG**  **(n = 254)** | | **Diabetes**  **(n = 61)** | ***P*** | **NGT**  **(n = 691)** | **IFG**  **(n = 153)** | **Diabetes**  **(n = 32)** | ***P*** | **NGT**  **(n = 1,053)** | **IFG**  **(n = 101)** | **Diabetes  (n = 29)** | ***P*** |
| Age (years) | 35.0±5.4^a^ | 38.1±4.6 | | 38.5±4.2 | **<0.001** | 35.2±5.5^a^ | 38.2±4.4 | 39.3±3.9 | **<0.001** | 34.9±5.3^a^ | 37.8±4.9 | 37.6±4.5 | **<0.001** |
| Height (cm) | 165.2±8.4^a^ | 167.5±8.6 | | 166.8±9.2 | **<0.001** | 172.9±5.6 | 172.7±6.1 | 173.5±6.3 | 0.766 | 160.1±5.4 | 159.7±5.4 | 159.5±5.7 | 0.688 |
| Weight (cm) | 62.6±12.0^a^ | 72.2±13.8 | | 74.2±16.7 | **<0.001** | 72.0±10.5^a^ | 77.7±12.0 | 80.2±11.4 | **<0.001** | 56.4±8.5^a^ | 63.9±12.0 | 67.6±13.1 | **<0.001** |
| BMI (kg/m^2^) | 22.8±3.3^a^ | 25.6±3.8 | | 26.6±4.1 | **<0.001** | 24.0±3.2^a^ | 26.0±3.3 | 26.6±3.6 | **<0.001** | 22.0±3.2^a^ | 25.0±4.3 | 26.6±4.6 | **<0.001** |
| WC (cm) | 77.3±9.5^a^ | 85.5±10.8 | | 87.4±10.3 | **<0.001** | 82.7±8.6^a^ | 88.3±9.6 | 89.7±9.6 | **<0.001** | 73.7±8.4^a^ | 81.2±11.0 | 84.9±10.5 | **<0.001** |
| SBP (mmHg) | 108.2±12.5^a^ | 116.7±13.9 | | 116.5±13.5 | **<0.001** | 113.4±12.0^a^ | 119.3±14.8 | 122.0±12.2 | **<0.001** | 104.8±11.6^a^ | 112.9±11.4 | 110.5±12.6 | **<0.001** |
| DBP (mmHg) | 71.7±10.1^a^ | 78.0±10.5 | | 79.5±10.5 | **<0.001** | 76.3±10.2^a^ | 81.1±10.3 | 83.9±9.8 | **<0.001** | 68.7±8.9^a^ | 73.4±8.8 | 74.7±9.2 | **<0.001** |
| TC (mg/dL) | 181.1±33.2^a^ | 194.1±36.7 | | 197.5±37.5 | **<0.001** | 188.0±34.9^a^ | 200.0±39.7 | 193.9±35.6 | **0.001** | 176.2±31.2^a^ | 185.1±29.7^b^ | 201.6±39.8 | **<0.001** |
| TG (mg/dL)^c^ | 86.5 (61.0-131.0)^a^ | 131.1 (86.0-206.0) | | 155.0 (100.8-246.0) | **<0.001** | 117.0 (80.0-172.0)^a^ | 118.0 (91.4-264.5) | 206.0 (112.0-272.0) | **<0.001** | 73.0 (54.0-105.0)^a^ | 86.0 (61.5-140.5)^b^ | 117.0 (94.0-183.0) | **<0.001** |
| HDL (mg/dL)^c^ | 48.7 (42.6-57.4)^a^ | 45.2 (37.3-53.9) | | 45.6 (39.5-51.5) | **<0.001** | 45.2 (39.1-52.2)^a^ | 43.4 (36.5-49.5) | 42.6 (38.2-47.8) | **0.001** | 52.2 (45.2-59.1)^a^ | 51.3 (42.6-59.6) | 46.9 (41.7-54.8) | **0.011** |
| LDL (mg/dL) | 109.1±28.6^a^ | 115.8 ± 31.7 | | 115.7±35.4 | **0.002** | 114.3±30.3 | 117.6±32.7 | 107.2±35.5 | 0.216 | 105.8±27.0 | 113.2±30.3 | 125.2±33.5 | **<0.001** |
| AST (IU/L)^c^ | 18.0 (15.0-21.0)^a^ | 20.0 (17.0-24.3)^b^ | | 22.5 (17.8-30.3) | **<0.001** | 20.0 (18.0-25.0)^a^ | 22.0 (19.0-27.0) | 25.0 (22.0-34.0) | **<0.001** | 16.0 (15.0-19.0)^a^ | 17.0 (15.0-21.0)^b^ | 18.0 (16.0-26.0) | **<0.001** |
| ALT (IU/L)^c^ | 15.0 (11.0-22.0)^a^ | 21.0 (15.0-33.0)^b^ | | 27.0 (18.8-48.0) | **<0.001** | 22.0 (16.0-31.0)^a^ | 27.0 (19.0-40.0)^b^ | 32.0 (27.0-54.0) | **<0.001** | 12.0 (10.0-15.0)^a^ | 15.0 (11.0-20.5)^b^ | 19.0 (12.0-31.0) | **<0.001** |
| GGT (IU/L)^c^ | 17.0 (13.0-28.0)^a^ | 31.0 (19.8-55.0) | | 40.0 (21.8-72.0) | **<0.001** | 29.0 (20.0-49.0)^a^ | 42.0 (30.0-70.0) | 63.0 (42.0-93.0) | **<0.001** | 13.0 (11.0-17.0)^a^ | 17.0 (14.0-26.0) | 22.0 (16.0-29.0) | **<0.001** |
| FPG (mg/dL) | 88.0±5.8^a^ | 105.8 ± 5.9^b^ | | 157.7±64.9 | **<0.001** | 89.3±5.7^a^ | 106.2±6.1^b^ | 165.9±60.4 | **<0.001** | 87.3±5.7^a^ | 105.3±5.6^b^ | 148.3±69.5 | **<0.001** |
| Insulin (μIU/mL)^c^ | 9.3 (7.6-11.5)^a^ | 11.2 (9.1-14.5) | | 11.8 (9.1-14.8) | **<0.001** | 9.5 (7.7-11.7)^a^ | 10.7 (8.9-14.5) | 11.0 (9.3-17.0) | **<0.001** | 9.1(7.6-11.4)^a^ | 12.0(9.1-14.7) | 12.0(7.6-13.8) | **<0.001** |
| HOMA-IR^c^ | 2.0(1.6-2.5)^a^ | 3.0(2.3-3.8)^b^ | | 4.1(3.1-5.7) | **<0.001** | 2.1(1.7-2.6)^a^ | 2.8(2.3-3.7)^b^ | 4.6(3.3-5.7) | **<0.001** | 2.0(1.6-2.5)^a^ | 3.1(2.3-3.9) | 3.6(2.5-6.0) | **<0.001** |
| HOMA-β^c^ | 136.8 (109.4-172.8)^a^ | 96.6 (77.7-124.8)^b^ | | 61.0 (31.0-116.2) | **<0.001** | 131.3 (105.5-165.5)^a^ | 94.6 (75.2-121.1)^b^ | 49.4 (31.7-83.6) | **<0.001** | 139.4 (112.6-175.9)^a^ | 101.6 (81.3-130.1)^b^ | 81.3 (28.8-120.6) | **<0.001** |
|  | | | | | | | | | | | | | |
| Family history of DM (n (%)) | | | | | | | | | | | | | |
| Any first degree relatives | 386 (22.1%) | 70 (27.6%) | 33 (54.1%) | | **<0.001** | 158 (22.9%) | 42 (27.5%) | 21 (65.6%) | **<0.001** | 228 (21.7%) | 28 (27.7%) | 12 (41.4%) | **0.006** |
| Father | 207 (11.9%) | 30 (11.8%) | 18 (29.5%) | | **<0.001** | 80 (11.6%) | 17 (11.1%) | 11 (34.4%) | **<0.001** | 127 (12.1%) | 13 (12.9%) | 7 (24.1%) | 0.056 |
| Mother | 132 (7.6%) | 30 (11.8%) | 11 (18.0%) | | **<0.001** | 59 (8.5%) | 19 (12.4%) | 7 (21.9%) | **0.001** | 73 (6.9%) | 11 (10.9%) | 4 (13.8%) | **0.023** |
| Both parents | 38 (2.2%) | 8 (3.1%) | 2 (3.3%) | | 0.120 | 18 (2.6%) | 5 (3.3%) | 1 (3.1%) | 0.386 | 20 (1.9%) | 3 (3.0%) | 1 (3.4%) | 0.242 |

Subjects aged 25 to 44 years from the Korean National Health and Nutrition Examination Survey 2010 are included in the analysis (n = 2,059). Data are presented as mean ± standard deviation (for normal distribution) or as median (interquartile range). *P*-values are calculated by ANOVA. The *post hoc* test was conducted using the Tukey’s method.

^a^*P* < 0.05 vs. IFG; ^b^*P* < 0.05 vs. DM; ^c^These variables were log transformed for the analysis.

**Supplemental Table 2.** Metabolic healthy status by family history of diabetes

|  |  | Total | | | Men | | | Women | |
| --- | --- | --- | --- | --- | --- | --- | --- | --- | --- |
|  |  | FH- | FH+ | FH- | | FH+ | FH- | | FH+ |
| Metabolic status | MHNO | 1107 (70.5%) | 301 (61.6%) | 371 (56.6%) | | 98 (44.3%) | 736 (80.4%) | | 203 (75.7%) |
|  | MHO | 274 (17.5%) | 86 (17.6%) | 159 (24.3%) | | 50 (22.6%) | 115 (12.6%) | | 36 (13.4%) |
|  | MUNO | 31 (2.0%) | 29 (5.9%) | 20 (3.1%) | | 17 (7.7%) | 11 (1.2%) | | 12 (4.5%) |
|  | MUO | 158 (10.1%) | 73 (14.9%) | 105 (16.0%) | | 56 (25.3%) | 53 (5.8%) | | 17 (6.3%) |
| Insulin resistance | HOMA-IR <2.5 | 1083 (69.0%) | 292 (59.7%) | 421 (64.3%) | | 113 (51.1%) | 662 (72.3%) | | 179 (66.8%) |
|  | HOMA-IR ≥2.5 | 487 (31.0%) | 197 (40.3%) | 234 (35.7%) | | 108 (48.9%) | 253 (27.7%) | | 89 (33.2%) |

Metabolically healthy when <3 components of metabolic syndrome are met.

MHNO, metabolically healthy non-obese; MHO, metabolically healthy obese; MUNO, metabolically unhealthy non-obese; MUO, metabolically unhealthy obese.

**Supplemental Table 3.** Subgroup analysis according to BMI

|  |  | FH- (n = 1,570) | FH+ (n = 489) | *P* |
| --- | --- | --- | --- | --- |
| Normal weight (n=1,062) | NGT | 789 (94.4%) | 203 (89.8%) | 0.014 |
|  | AGT | 47 (5.6%) | 23 (10.2%) |  |
|  | MS- | 827 (98.9%) | 219 (96.9%) | 0.027 |
|  | MS+ | 9 (1.1%) | 7 (3.1%) |  |
| Overweight (n=406) | NGT | 256 (84.8%) | 81 (77.9%) | 0.107 |
|  | AGT | 46 (15.2%) | 23 (22.1%) |  |
|  | MS- | 280 (92.7%) | 82 (78.8%) | <0.001 |
|  | MS+ | 22 (7.3%) | 22 (21.2%) |  |
| Obese  (n=591) | NGT | 313 (72.5%) | 102 (64.2%) | 0.050 |
|  | AGT | 119 (27.5%) | 57 (35.8%) |  |
|  | MS- | 274 (63.4%) | 86 (54.1%) | 0.039 |
|  | MS+ | 158 (36.6%) | 73 (45.9%) |  |

Normal weight: BMI < 23 kg/m^2^, overweight: 23 ≤ BMI < 25 kg/m^2^, obese: BMI ≥ 25 kg/m^2^.

AGT, abnormal glucose tolerance: either impaired fasting glucose or diabetes.

MS, metabolic syndrome: ≥3 of the criteria are met.

**Supplemental Table 4.** Clinical characteristics of non-diabetic subjects according to family history of T2DM in first-degree relatives.

|  | **FH- (n = 1,542)** | **FH+ (n = 456)** | ***P*** | ***P***^a^ | ***P***^b^ |
| --- | --- | --- | --- | --- | --- |
| Age (years) | 35.2±5.4 | 36.1±5.1 | **0.001** |  |  |
| Male, n (%) | 644 (41.8%) | 200 (43.9%) | 0.426 |  |  |
| Height (cm) | 165.3±8.5 | 165.9±8.3 | 0.216 | 0.140 | 0.306 |
| Weight (cm) | 63.4±12.6 | 65.3±12.9 | **0.006** | **0.009** | 0.285 |
| BMI (kg/m^2^) | 23.1±3.5 | 23.6±3.5 | **0.005** | **0.016** |  |
| WC (cm) | 78.1±10.1 | 79.2±9.8 | **0.032** | 0.090 | 0.506 |
| SBP (mmHg) | 108.9±13.0 | 110.6±12.9 | **0.016** | 0.054 | 0.225 |
| DBP (mmHg) | 72.1±10.4 | 73.8±10.3 | **0.002** | **0.012** | 0.064 |
| TC (mg/dL) | 181.5±34.1 | 186.8±33.2 | **0.003** | **0.012** | 0.051 |
| TG (mg/dL)^c^ | 89 (62.0-134.0) | 99 (64.3-160.8) | **<0.001** | **0.001** | **0.008** |
| HDL (mg/dL)^c^ | 48.7 (41.7-56.5) | 46.9 (40.8-56.5) | 0.056 | 0.148 | 0.421 |
| LDL (mg/dL) | 109.3±29.5 | 112.3±27.8 | 0.052 | 0.188 | 0.321 |
| AST (IU/L)^c^ | 18 (15.0-21.0) | 18 (16.0-22.0) | 0.318 | 0.516 | 0.995 |
| ALT (IU/L)^c^ | 15 (11.0-23.0) | 16 (12.0-25.0) | **0.049** | 0.088 | 0.461 |
| GGT (IU/L)^c^ | 17 (13.0-30.0) | 20 (14.0-33.0) | **0.024** | 0.067 | 0.302 |
| FPG (mg/dL) | 89.9±8.2 | 91.7±8.5 | **<0.001** | **<0.001** | **0.005** |
| Insulin (μIU/mL)^c^ | 9.4(7.8-11.6) | 9.5(7.8-12.4) | 0.141 | 0.129 | 0.602 |
| HOMA-IR^c^ | 2.1(1.7-2.6) | 2.2(1.7-2.8) | **0.021** | **0.030** | 0.234 |
| HOMA-β^c^ | 133.3 (104.5-170.7) | 127.4 (100.3-160.0) | **0.036** | 0.143 | 0.063 |

Subjects with normal glucose tolerance and impaired fasting glucose are included in this analysis. Data are presented as mean ± standard deviation (for normal distribution) or as median (interquartile range). *P*-values are calculated by *t*-test.

^a^*P*-values adjusted for age; ^b^*P*-values adjusted for age and BMI; ^c^These variables were log transformed for the analysis.

**Supplemental Table 5.** Number of family members with diabetes and the risk of abnormal glucose tolerance and metabolic syndrome

| Number of family members with diabetes | Abnormal glucose tolerance | | Metabolic syndrome | |
| --- | --- | --- | --- | --- |
|  | n (%) | Odds ratio | n (%) | Odds ratio |
| 0 (n = 1,570) | 212 (13.5%) | 1 (ref.) | 189 (12.0%) | 1 (ref.) |
| 1 (n = 421) | 86 (20.4%) | 1.64 (1.25 - 2.17) | 83 (19.7%) | 1.79 (1.35 - 2.38) |
| 2+ (n = 68) | 17 (25.0%) | 2.14 (1.21 - 3.77) | 19 (27.9%) | 2.83 (1.63 - 4.92) |

Data are calculated by binary logistic regression analysis and presented as odds ratio (95% confidence interval). Abnormal glucose tolerance refers to either having impaired fasting glucose or diabetes.

**Supplemental Table 6. Exercise and dietary pattern by family history of diabetes**

|  | FH (-) (n = 1570) | FH (+) (n = 489) | *P* |
| --- | --- | --- | --- |
| ***Exercise*** |  |  |  |
| Regular exercise^a^ | 309 (19.7%) | 89 (18.2%) | 0.469 |
| Trying to lose weight^b^ | 322 (74.5%) | 116 (73.0%) | 0.697 |
| ***Diet*** |  |  |  |
| Energy intake (kcal/day) | 2149.1 ± 934.1 | 2174.8 ± 1048.7 | 0.636 |
| Protein (g) | 79.7 ± 43.7 | 83.8 ± 57.6 | 0.129 |
| Fat (g) | 49.6 ± 33.1 | 51.9 ± 41.4 | 0.239 |
| Carbohydrate (g) | 329.0 ± 125.5 | 328.1 ± 133.4 | 0.898 |

^a^ Regular exercise was defined as moderate or vigorous intensity physical activities.

^b^ Subjects with BMI over 25 are included for this analysis (n = 591).
